# Circulating acetylcholine serves as a potential biomarker role in pulmonary hypertension

**DOI:** 10.1186/s12890-024-02856-7

**Published:** 2024-01-16

**Authors:** Yicheng Yang, Jing Xu, Songren Shu, Peizhi Wang, Yanru Liang, Bingyang Liu, Beilan Yang, Hanwen Zhang, Qing Zhao, Zhihui Zhao, Qin Luo, Zhihong Liu, Qixian Zeng, Changming Xiong

**Affiliations:** 1 Respiratory and Pulmonary Vascular Center, State Key Laboratory of Cardiovascular Disease, Fuwai Hospital, National Center for Cardiovascular Diseases, Chinese Academy of Medical Sciences and Peking Union Medical College, North Lishi Road, Xicheng District, No. 167, Beijing, 100037 China; 2 Department of Cardiology, State Key Laboratory of Cardiovascular Disease, Fuwai Hospital, National Center for Cardiovascular Diseases, Chinese Academy of Medical Sciences and Peking Union Medical College, Beijing, 100037 China; 3 Department of Cardiac Surgery, State Key Laboratory of Cardiovascular Disease, Fuwai Hospital, National Center for Cardiovascular Diseases, Chinese Academy of Medical Sciences, Peking Union Medical College, Beijing, 100037 China

**Keywords:** Acetylcholine, Metabolite, Pulmonary hypertension, Biomarker, Prognosis

## Abstract

**Background:**

An increased acetylcholine (ACh) level in the right ventricle tissue of pulmonary hypertension (PH) was revealed, which indicated the important role of ACh in disease pathogenesis. However, the relationship between plasma ACh levels and disease conditions and patients’ prognosis has not been investigated. We aimed to explore the association between plasma ACh levels and the prognosis of patients with PH. We also discussed the feasibility of plasma ACh as a biomarker, which may contribute to the management of PH patients in the future.

**Methods:**

Patients with confirmed PH in Fuwai Hospital from April 2019 to August 2020 were enrolled. The primary clinical outcome in this study was defined as a composite outcome, including death/lung transplantation, heart failure, and worsening of symptoms. Fasting plasma was collected to detect the ACh levels. The association between ACh levels and patients’ prognosis was explored.

**Results:**

Finally, four hundred and eight patients with PH were enrolled and followed for a mean period of 2.5 years. Patients in the high ACh group had worse World Health Organization Functional Class (WHO-FC), lower 6-minute walk distance (6 MWD), and higher N-terminal pro-brain natriuretic peptide (NT-proBNP). Notably, echocardiographic and hemodynamic parameters in the high metabolite group also suggested a worse disease condition compared with the low ACh group. After adjusting for confounders, compared with low ACh patients, those with high metabolite levels still have worse prognoses characterized as elevated risk of mortality, heart failure, and symptoms worsening.

**Conclusion:**

High circulating ACh levels were associated with severe PH conditions and poor prognosis, which might serve as a potential biomarker in PH.

**Supplementary Information:**

The online version contains supplementary material available at 10.1186/s12890-024-02856-7.

## Introduction

Pulmonary hypertension (PH) is a life-threatening cardiovascular disease characterized by a chronic and progressive increase in pulmonary vascular resistance (PVR), leading to remodelling of the right ventricle and eventual right ventricular failure [1]. Even though the management of PH has improved significantly since a decade ago, the prognosis of patients is still far from satisfactory. The symptoms of PH present nonspecifically like many other lung diseases including dyspnea and fatigue, which often cause a delay in clinical diagnosis and optimal therapy.

Acetylcholine (ACh) is a well-established signalling molecule that serves as the primary chemical neurotransmitter regulating various physiological functions [2]. In the heart, ACh is released through the parasympathetic branch of the autonomous nervous system as a neuronal source [3] but is also synthesized and released from nonneuronal cells, including cardiomyocytes [4]. The ACh-mediated cholinergic system plays a crucial role in modulating the intricate interactions between sympathetic and parasympathetic responses, thereby maintaining the homeostasis of cardiac physiology [5]. In general, parasympathetic stimulation tends to counterbalance the actions of the sympathetic nervous system, leading to a reduction in heart rate, atrial contractility, and conduction velocities of the sinoatrial and atrioventricular nodes [6]. Recent advancements have highlighted the protective effects of ACh in the heart under several pathologic conditions, such as sympathetic hyperactivity-induced hypertrophy [7], myocardial infarction [8], hypertension [9], cardiomyopathy [10], and ventricular dysfunction [11]. Additionally, ACh’s cardioprotective actions have been associated with anti-inflammatory responses, revealing an unrecognized beneficial function of ACh in the cardiovascular system [5]. The effects of ACh on pulmonary circulation have been investigated by previous works [12–14]. α7 nicotinic acetylcholine receptor (α7 nAChR) plays an essential role in the execution of biological functions of acetylcholine. Activation of α7 nAChR by acetylcholine may promote the right ventricular remodelling and PH [15]. A recent study has demonstrated an increased ACh level in the right ventricle tissue of PH, indicating its potentially important role in disease pathogenesis [16]. However, to date, the relationship between plasma ACh levels and disease conditions and PH patients’ prognosis has not been elucidated.

In this study, our objective was to investigate the relationship between plasma ACh levels and the severity and prognosis of patients with PH, as well as to discuss the potential of plasma ACh as a biomarker for the future management of PH patients.

## Methods

This clinical study was approved by the ethics committee of Fuwai Hospital and adhered to the Declaration of Helsinki. All the patients enrolled in this study provided written informed consent.

### Study population

PH was defined as a mean pulmonary arterial pressure (mPAP) ≥ 25 mmHg at resting by right heart catheterisation according to the 2015 European Society of Cardiology PH guideline [17]. Although the threshold for the diagnosis of PH was reduced to 20 mmHg in the latest PH guideline [18], we used the previous criteria in this study because the new threshold had not been recommended by the European Society of Cardiology when the cohort started. Inpatients with confirmed PH in Fuwai Hospital, National Cardiovascular Center from April 2019 to August 2020 were enrolled. Exclusion criteria included individuals with tumours, nervous system diseases, and pregnant women. Those who declined to participate in the study and lost to follow-up were also excluded.

### Clinical data collection

Fasting plasma samples were used for the measurement of acetylcholine and other laboratory indicators. Hemodynamic parameters including mean right atrial pressure (mRAP), mPAP, pulmonary arterial wedge pressure (PAWP), PVR, and cardiac output index were obtained by right heart catheterisation. Demographic characteristics, body mass index (BMI, calculated as weight/height^2^), World Health Organization Functional Class (WHO-FC), 6-minute walk distance (6 MWD), and echocardiographic data were also collected from the electronic medical records.

### Clinical outcome and follow-up

The primary clinical outcome in this study was defined as a composite outcome including death/lung transplantation, heart failure, and worsening of symptoms. The secondary endpoint was considered as death/lung transplantation, heart failure, and worsening of symptoms, respectively. Patients were followed up through the outpatient department, rehospitalization, or telephone call. Follow-up duration was defined from the first time of ACh measurement to the occurrence of outcomes or the end of follow-up (September 2022).

### Statistical analysis

A restricted cubic spline was used to illustrate the association between ACh levels and clinical outcomes and to explore the cut-off value of circulating ACh levels. PH patients were divided into two groups based on the metabolite levels and differences were analysed using Student’s t-tests or nonparametric test and chi-square tests for categorical variables. Kaplan–Meier (KM) analysis and Cox proportional hazards regression were used for survival analyses. Propensity score matching was used to adjust for the potential confounders aiming to further reveal the effects of circulating ACh levels on PH prognosis. A two-sided *P*-value of less than 0.05 was considered statistical significance. Statistical analyses were performed using R 2.8.0 (Vienna, Austria) and SPSS (version 23; IBM Corp, 2015).

## Results

### Patient cohort

A total of 408 patients with PH were enrolled and followed for an average of 2.5 (2.5 ± 0.9) years in this cohort study. Briefly, we included a total of 294 patients with pulmonary arterial hypertension (Group 1), 106 patients with PH in Group 4, and 8 patients with other subtypes of PH. During the follow-up, 133 individuals had primary outcomes including 58 death, 106 heart failure, and 130 symptoms worsening.

### Basic characteristics of PH patients stratified by circulating ACh levels

**Fig. 1:**
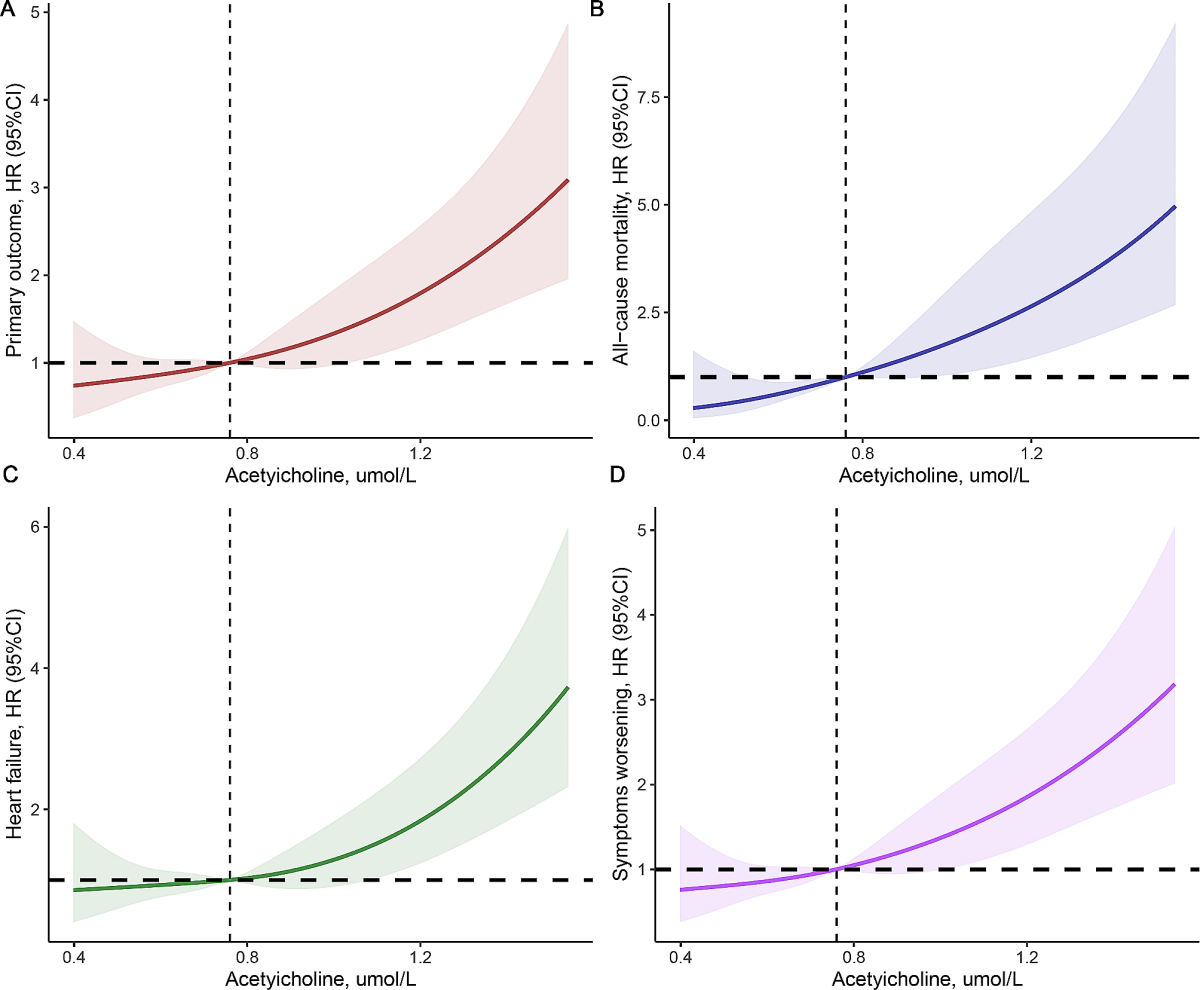
Restricted cubic spline result of Acetylcholine levels with hazard ratios for the risk of primary outcome (**A**), death (**B**), heart failure (**C**), and symptoms worsening (**D**). HR: hazard ratio; CI: confidence interval

Figure 1A illustrates the association between circulating ACh levels and the risk of composite clinical outcomes. When the level of ACh was higher than 0.76 µmol/L, the risk of adverse events increased among PH patients. Similar trends were explored between metabolite with death, heart failure, and symptoms worsening, as displayed in Fig. 1B-D.

Patients with PH were divided into two cohorts based on the ACh levels (with a cut-off value of 0.76 µmol/L). Table 1 shows the basic clinical characteristics of patients. Briefly, patients were older, male-dominated, and with elevated creatinine levels in the high ACh group. Compared with those in the low metabolite group, patients stratified in the high ACh group had worse WHO-FC, lower 6 MWD, and higher N-terminal pro-brain natriuretic peptide (NT-proBNP). Notably, echocardiographic and hemodynamic parameters in the high metabolite group indicate lower tricuspid annular plane systolic excursion (TAPSE) and cardiac index, as well as higher mRAP and PVR, suggesting a more severe disease condition compared with the low ACh group. There were no significant differences in treatment between the groups, except for the use of endothelin receptor inhibitors.

**Table 1:** Baseline characteristics of patients with PH stratified by acetylcholine levels

Variables	Total PH patients N = 408	Low acetylcholine N = 205	High acetylcholine N = 203
Age, years	43.1 ± 16.6	38.3 ± 14.9	48.0 ± 16.8*
Female sex, n (%)	278 (68.1)	173 (84.4)	105 (51.7)*
BMI, kg/m2	22.1 ± 3.9	22.1 ± 4.2	22.2 ± 3.5
6 MWD, m	385.0 ± 120.0	407.4 ± 110.7	363.2 ± 124.8*
WHO-FC, n (%)			
I-II	259 (63.5)	143 (69.8)	116 (57.1)*
III	116 (28.4)	49 (23.9)	67 (33.0)*
IV	33 (8.1)	13 (6.3)	20 (9.9)#
Laboratories			
Acetylcholine, µmol/L	0.8 ± 0.3	0.6 ± 1.1	1.0 ± 0.3*
NT-proBNP, pg/ml	585.4 (161.5, 1702.3)	395.3 (116.4, 1152.5)	881.0 (278.7, 2258.0)*
Albumin, g	42.0 ± 5.0	42.4 ± 4.6	41.5 ± 5.4
Creatinine, µmol/L	79.5 ± 23.4	70.5 ± 15.0	88.5 ± 26.8*
Echocardiography			
LVEF, %	64.8 ± 6.9	64.8 ± 7.8	64.7 ± 5.8
RVD, mm	32.4 ± 7.6	31.5 ± 7.3	33.4 ± 7.9#
TAPSE, mm	16.6 ± 4.1	17.1 ± 4.2	16.3 ± 4.2#
Hemodynamics			
mRAP, mmHg	6.0 (3.0, 9.0)	5.0 (3.0, 8.0)	7.0 (4.0, 9.0)*
mPAP, mmHg	55.5 ± 17.1	50.6 ± 16.7	53.9 ± 14.3
Cardiac index, L/min*m2	3.1 ± 0.9	3.3 ± 1.0	2.8 ± 0.8*
PVR, Wu	10.0 ± 5.1	9.4 ± 5.3	10.7 ± 5.0#
PAWP, mmHg	8.2 ± 4.0	8.0 ± 4.2	8.5 ± 3.9
Treatment, n (%)			
PDE5i	274 (67.2)	143 (69.8)	131 (64.5)
ERAs	226 (55.4)	126 (61.5)	100 (49.3)#
Prostacyclins	56 (13.7)	24 (11.7)	32 (15.8)
Riociguat	35 (8.6)	14 (6.8)	21 (10.3)
BPA/PEA	35 (8.6)	14 (6.8)	21 (10.3)

Patients were divided into two groups based on the levels of plasma acetylcholine (the cut-off value was 0.76 µmol/L). According to the different data distributions, continuous variables were presented as mean ± standard deviation or median and interquartile ranges. Categorical variables were shown as frequencies with percentages. Student’s t-test or Wilcoxon rank-sum test was utilized for continuous data while Chi-square test was for categorical variables

PH: pulmonary hypertension; BMI: body mass index; 6 MWD: 6-minute walk distance; WHO-FC: world health organization function class; NT-proBNP: N-terminal pro-brain natriuretic peptide; LVEF: left ventricular ejection fraction; RVD: right ventricular diameter; TAPSE: tricuspid annular plane systolic excursion; mRAP: mean right atrial pressure; mPAP: mean pulmonary arterial pressure; PVR: pulmonary vascular resistance; PAWP: pulmonary arterial wedge pressure; PDE5i: phosphodiesterase type 5 inhibitor; ERAs: endothelin receptor agonists; BPA: balloon pulmonary angioplasty; PEA: pulmonary endarterectomy

*P < 0.001; ^#^*P* < 0.05

### High ACh levels were associated with poor prognosis

**Fig. 2:**
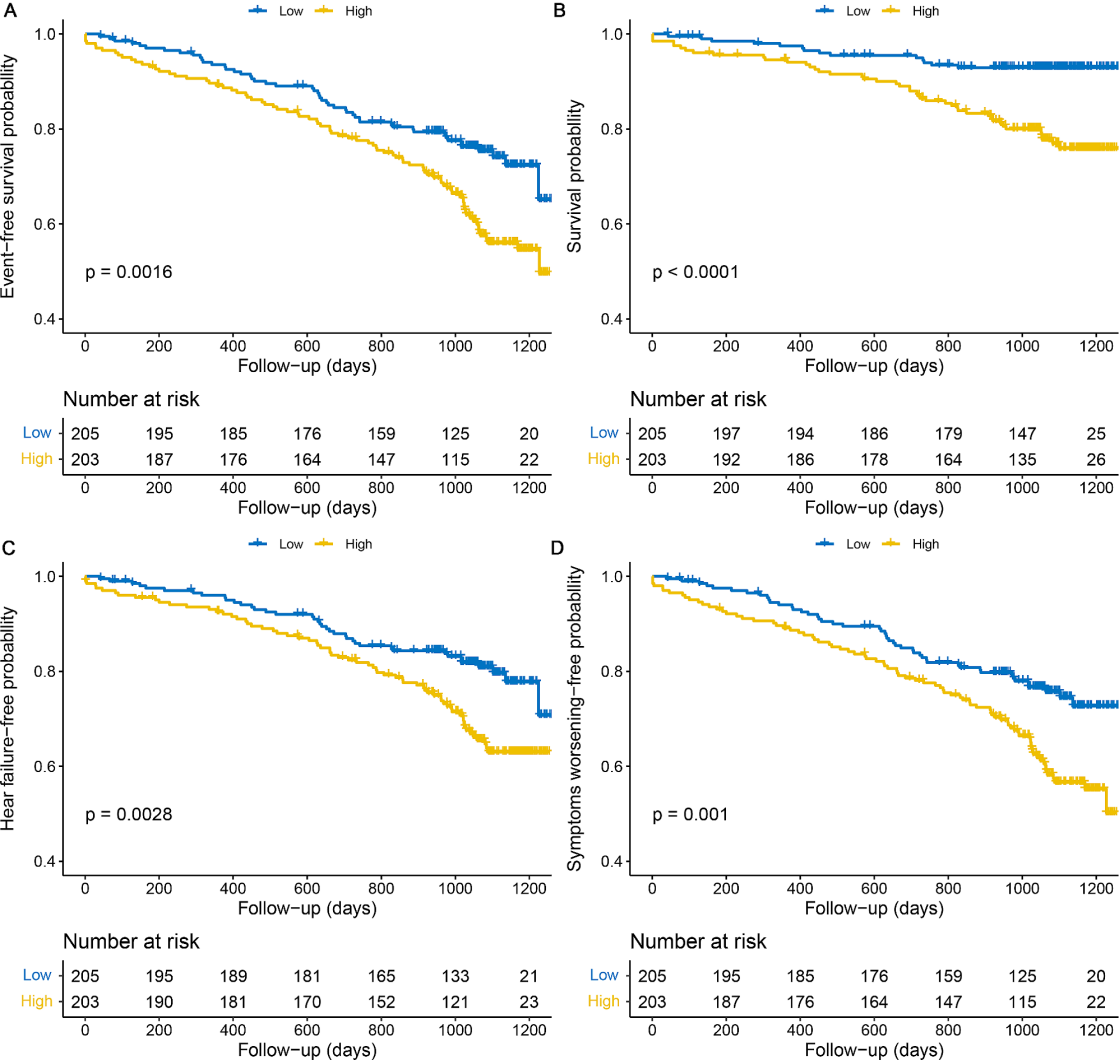
Kaplan-Meier analysis for the incidence of composite outcome events (**A**), death (**B**), heart failure (**C**), and symptoms worsening (**D**) in patients with high and low acetylcholine levels. Four hundred and eight patients with PH were analyzed (n = 203 in the high acetylcholine group; n = 205 in the low acetylcholine group). *P*-value calculated by the log-rank test

Patients in the high ACh group were more likely to experience a poor prognosis than those in the low group (Fig. 2). The estimated incidences of both composite events (*P* = 0.0016, Fig. 2A) and secondary clinical outcomes (Fig. 2B-D) differed significantly between the two cohorts. Supplementary Table 1 shows the results of univariate Cox regression analysis. After adjusting for the confounders including age, albumin, treatment, PH subtypes, and comorbidities, high metabolite levels remained significantly associated with the increased risk of adverse endpoint events [hazard ratio (HR) = 2.450, 95% CI: 1.414–4.247; *P* = 0.001]. Furthermore, even after adjusting for the factors which were strongly associated with the prognosis of PH, including 6 MWD, WHO-FC, and echocardiographic parameters, a similar trend was observed [HR = 1.958, 95% CI: 1.141–3.362; *P* = 0.015, Table 2].

**Table 2:** Multivariate cox analysis of acetylcholine and prognosis in patients with PH

Variables	HR	95% CI	*P* value
Unadjusted	3.733	2.264–6.156	< 0.001
Adjusted^a^	2.450	1.414–4.247	0.001
Adjusted^b^	1.958	1.141–3.362	0.015

PH: pulmonary hypertension; CI: confidence interval; 6 MWD: 6-minute walk distance; WHO-FC: world health organization function class; RVD: right ventricular diameter; TAPSE: tricuspid annular plane systolic excursion

^a^Adjusted for age, prostacyclins, PH subtypes, albumin, and comorbidities including coronary heart disease and chronic kidney disease

^b^Adjusted for 6 MWD, WHO-FC, and echocardiographic parameters including RVD and TAPSE

### Sensitivity analyses after propensity score matching

We noticed that patients were old, male-dominated, and with elevated creatinine levels in the high ACh group. Propensity score matching was used to reduce the confounding effects of these factors. In this phase, 252 PH patients were included for analysis (Supplementary Fig. 1) and the characteristics were shown in Supplementary Table 2. Our findings consistently demonstrated that elevated ACh levels remained associated with a poor prognosis for patients with PH, aligning with previous results.

## Discussion

Despite significant advancements in therapy options and treatment strategies for PH, improper management and inadequate evaluation remain common issues. Given the progressive nature of PH, risk stratification and timely ascertaining the severity are crucial for adjusting treatment and reducing serious consequences. The exploration of effective plasma biomarkers is essential for disease management.

In this study, we identified the underlying connection between the circulating ACh level and the risk of poor prognosis using the restricted cubic spline analysis. Our findings revealed that higher ACh levels correlated with more severe baseline characteristics and worse clinical manifestations in PH patients, including worse WHO-FC, lower 6 MWD, and higher NT-proBNP levels, as well as worse echocardiographic and hemodynamic parameters. These results align with findings of a recent work that increased ACh is significantly associated with right ventricular fibrosis and dysfunction by activation of α7 nAChR [16]. It has been shown that activation of α7 nAChR contributes to the abnormal proliferation of vascular smooth muscle cells and adventitial fibroblasts [19], as well as accelerating neovascularization and fibrovascular growth [20]. Notably, α7 nAChR stimulation may lead to increased pulmonary blood pressure accompanied by right ventricular remodelling, inducing progressive and persistent PH [15]. Hence, it is plausible that that high ACh levels may participate in the PH pathogenesis through the overactivation of α7 nAChR signalling, leading to maladaptive vascular fibrosis and ventricular dysfunction. Additional studies will be required to identify the molecular mechanisms and investigate the potential clinical significance of targeting plasma ACh levels in PH patients to improve symptoms and outcomes.

Furthermore, our study demonstrated a strong association between high ACh levels and poor prognosis, which remained significant even after propensity score matching to reduce the effects of confounding factors. A possible explanation for this might be the feedback regulation of cholinergic transdifferentiation and ACh production. The autonomic nervous system, especially the sympathetic/parasympathetic balance, is a crucial regulator of cardiovascular function [21, 22]. Research has implicated that the overactivation of the sympathetic nervous system contributes to cardiac remodelling and blood pressure elevation, which is usually associated with poorer prognosis and higher morbidity and mortality [23–25]. In chronic PH, increased cholinergic tone and induction of ACh production may counteract sympathetic action and help restore autonomic function to maintain heart homeostasis [26]. Oral administration of donepezil, an acetylcholinesterase inhibitor, could markedly improve the long-term survival of chronic heart failure rats by preventing ventricular dysfunction and cardiac remodelling [27]. Studies have shown that increasing Ach level by acetylcholinesterase inhibition via pyridostigmine could reduce pulmonary vascular resistance, right ventricular afterload, and pulmonary vascular remodelling, coupling with reduced local and systemic inflammation [28]. The cholinergic anti-inflammatory pathway, involving both vagal and nonneuronal ACh, has been found to have cardioprotective effects in heart failure [29]. Based on the previous knowledge, we speculate that circulating ACh may be a response to PH and could potentially have a beneficial effect on preventing disease progression. These findings highlight the complex role ACh responses might play in regulating cardiovascular function in health and disease via multiple receptors and pathways. Importantly, our results indicated the potential use of ACh as a plasma-based biomarker to monitor PH progression, but further confirmation is needed through large-scale prospective cohort studies.

Although this study was conducted with a rigorous design, there were still several limitations. It was a single-center study and the results needed to be verified in multi-center research. In addition, the specific role of ACh in PH pathogenesis was still unknown and basic research was warranted to reveal the inner association. Nevertheless, our study first demonstrated the potential biomarker role of ACh in PH indicating its value in disease management.

## Conclusion

High circulating ACh levels were associated with severe PH conditions. Compared with low ACh patients, those with high metabolite levels had worse prognoses characterized by an elevated risk of mortality, heart failure, and symptoms worsening. This indicates that circulating ACh may serve as a potential biomarker in PH.

### Electronic supplementary material

Below is the link to the electronic supplementary material.

Supplementary Table S1: Univariate Cox regression analysis of variables

Supplementary Table S2: Baseline characteristics of patients with PH stratified by acetylcholine levels after propensity score matching

Supplementary Fig. S1: Kaplan-Meier analysis for the incidence of composite outcome events(A), death (B), heart failure (C), and symptoms worsening (D) in patients with high and low acetylcholine levels after propensity score matching. Two hundred and fifty two patients with PH were analyzed (n=126 in high acetylcholine group; n=126 in low acetylcholine group). P-value calculated by the log-rank test

## Data Availability

The datasets analysed during the current study are not publicly available due to [REASON WHY DATA ARE NOT PUBLIC] but are available from the corresponding author upon reasonable request.

## Abbreviations

ACh: Acetylcholine
PVR: Pulmonary vascular resistance
mPAP: mean pulmonary arterial pressure
mRAP: mean right atrial pressure
KM: Kaplan–Meier
