# Supplementary material for: Circulating acetylcholine serves as a potential biomarker role in pulmonary hypertension
Source: BMC Pulm Med. 2024 Jan 16;24:35. doi: 10.1186/s12890-024-02856-7 (PMC10792774; doi:10.1186/s12890-024-02856-7)
Supplement: Supplementary file 1 — Supplementary Table S1: Univariate Cox regression analysis of variables [file 12890_2024_2856_MOESM1_ESM.docx]

**Table S1. Univariate Cox regression analysis of variables**

| **Variable** | **HR** | **95% CI** | ***P*** |
| --- | --- | --- | --- |
| Age, year | 1.016 | 1.005**–**1.026 | **0.003** |
| Sex, female | 0.812 | 0.569**–**1.159 | 0.252 |
| BMI, kg/m^2^ | 0.961 | 0.917**–**1.008 | 0.100 |
| 6 MWD | 0.996 | 0.994**–**0.997 | **<0.001** |
| WHO-FC | 2.344 | 1.867**–**2.942 | **<0.001** |
| Acetylcholine, μmol/L | 3.733 | 2.264-6.156 | **<0.001** |
| Albumin, g | 0.911 | 0.888-0.935 | **<0.001** |
| Creatinine, μmol/L | 1.018 | 1.011-1.024 | **<0.001** |
| LVEF, % | 0.992 | 0.970**–**1.014 | 0.460 |
| RVD, mm | 1.044 | 1.023**–**1.067 | **<0.001** |
| TAPSE, mm | 0.902 | 0.865**–**0.940 | **<0.001** |
| mRAP, mmHg | 1.094 | 1.045**–**1.146 | **<0.001** |
| mPAP, mmHg | 1.002 | 0.990-1.014 | 0.774 |
| Cardiac index, L/(min*m^2^) | 0.608 | 0.464-0.796 | **<0.001** |
| PVR, Wu | 1.054 | 1.04-1.106 | **0.034** |
| PAWP, mmHg | 0.988 | 0.929-1.051 | 0.706 |
| Hypertension | 1.024 | 0.630-1.665 | 0.924 |
| Coronary heart disease | 2.502 | 1.461-4.286 | **0.001** |
| Chronic kidney disease | 2.107 | 1.070-4.147 | **0.031** |
| Diabetes | 1.677 | 0.904-3.110 | 0.101 |
| PDE5i | 0.862 | 0.605-1.408 | 0.411 |
| ERAs | 0.962 | 0.684-1.353 | 0.825 |
| Prostacyclins | 1.679 | 1.0860-2.596 | **0.020** |
| Riociguat | 1.094 | 0.617-1.940 | 0.758 |
| BPA/PEA | 0.657 | 0.321-1.343 | 0.249 |

CI: confidence interval; BMI: body mass index; 6 MWD: 6-minute walk distance; WHO-FC: world health organization function class; NT-proBNP: N-terminal pro-brain natriuretic peptide; LVEF: left ventricular ejection fraction; RVD: right ventricular diameter; TAPSE: tricuspid annular plane systolic excursion; mRAP: mean right atrial pressure; mPAP: mean pulmonary arterial pressure; PVR: pulmonary vascular resistance; PAWP: pulmonary artery wedge pressure; PDE5i: phosphodiesterase type 5 inhibitor; ERAs: endothelin receptor agonists; BPA: balloon pulmonary angioplasty; PEA: pulmonary endarterectomy.
