# Supplementary material for: Circulating acetylcholine serves as a potential biomarker role in pulmonary hypertension
Source: BMC Pulm Med. 2024 Jan 16;24:35. doi: 10.1186/s12890-024-02856-7 (PMC10792774; doi:10.1186/s12890-024-02856-7)
Supplement: Supplementary file 2 — Supplementary Table S2: Baseline characteristics of patients with PH stratified by acetylcholine levels after propensity score matching [file 12890_2024_2856_MOESM2_ESM.docx]

**Table S2. Baseline characteristics of patients with PH stratified by acetylcholine levels after propensity score matching**

| **Variables** | **Total PH patients**  **N=252** | **Low acetylcholine**  **N=126** | **High acetylcholine**  **N=126** |
| --- | --- | --- | --- |
| Age, years | 43.5±16.0 | 43.0±16.1 | 44.0±16.0 |
| Female sex, n (%) | 186 (73.8) | 96 (76.2) | 90 (71.4) |
| BMI, kg/m^2^ | 22.3±3.9 | 22.6±4.2 | 22.0±3.6 |
| 6 MWD, m | 388.8±123.3 | 410.4±112.1 | 368.5±130.2# |
| **WHO-FC, n (%)** |  |  |  |
| I-II | 162 (64.3) | 87 (69.0) | 75 (59.5)# |
| III | 66 (26.2) | 30 (23.8) | 36 (28.6) |
| IV | 24 (9.5) | 9 (7.1) | 15 (11.9)# |
| **Laboratories** |  |  |  |
| Acetylcholine, μmol/L | 0.8±0.3 | 0.6±0.1 | 1.0±0.2* |
| NT-proBNP, pg/ml | 661.9 (175.9, 1780.5) | 511.6 (150.4, 1414.5) | 895.4 (250.0, 2337.3)* |
| Albumin, g | 41.8±5.0 | 42.3±4.3 | 41.4±5.6 |
| Creatinine, μmol/L | 77.9±15.6 | 77.0±14.3 | 78.8±16.8 |
| **Echocardiography** |  |  |  |
| LVEF, % | 65.1±6.8 | 65.3±8.2 | 64.9±5.1 |
| RVD, mm | 32.2±7.3 | 31.9±7.4 | 32.7±7.3 |
| TAPSE, mm | 16.4±4.4 | 16.8±4.3 | 16.1±4.4 |
| **Hemodynamics** |  |  |  |
| mRAP, mmHg | 6.0 (3.0, 8.8) | 5.0 (2.0, 8.0) | 7.0 (4.0, 9.0)# |
| mPAP, mmHg | 56.4±17.4 | 54.3±17.7 | 58.6±16.9 |
| Cardiac index, L/min*m^2^ | 3.0±0.8 | 3.1±0.9 | 2.9±0.8 |
| PVR, Wu | 10.3±5.5 | 9.5±5.5 | 11.3±5.4 |
| PAWP, mmHg | 8.3±3.8 | 8.1±3.4 | 8.5±4.0 |
| **Treatment, n (%)** |  |  |  |
| PDE5i | 168 (66.7) | 86 (68.3) | 82 (65.1) |
| ERAs | 129 (51.2) | 67 (53.2) | 62 (49.2) |
| Prostacyclins | 38 (15.1) | 16 (12.7) | 22 (17.5) |
| Riociguat | 23 (9.1) | 11 (8.7) | 12 (9.5) |
| BPA/PEA | 23 (9.1) | 11 (8.7) | 12 (9.5) |

Patients were divided into two groups based on the levels of plasma acetylcholine (the cut-off value was 0.76 μmol/L). According to the different data distribution, continuous variables were presented as mean ± standard deviation or median and interquartile ranges. Categorical variables were shown as frequencies with percentages. Student's t-test or Wilcoxon rank-sum test was utilized for continuous data while Chi-square test was for categorical variables. PH: pulmonary hypertension; BMI: body mass index; 6 MWD: 6-minute walk distance; WHO-FC: world health organization function class; NT-proBNP: N-terminal pro-brain natriuretic peptide; LVEF: left ventricular ejection fraction; RVD: right ventricular diameter; TAPSE: tricuspid annular plane systolic excursion; mRAP: mean right atrial pressure; mPAP: mean pulmonary arterial pressure; PVR: pulmonary vascular resistance; PAWP: pulmonary artery wedge pressure; PDE5i: phosphodiesterase type 5 inhibitor; ERAs: endothelin receptor agonists; BPA: balloon pulmonary angioplasty; PEA: pulmonary endarterectomy. * ***P*** < 0.001; # ***P*** < 0.05
