# Supplementary material for: Circulating acetylcholine serves as a potential biomarker role in pulmonary hypertension
Source: BMC Pulm Med. 2024 Jan 16;24:35. doi: 10.1186/s12890-024-02856-7 (PMC10792774; doi:10.1186/s12890-024-02856-7)
Supplement: Supplementary file 3 — Supplementary Fig. S1: Kaplan-Meier analysis for the incidence of composite outcome events(A), death (B), heart failure (C), and symptoms worsening (D) in patients with high and low acetylcholine levels after propensity score matching. Two hundred and fifty two patients with PH were analyzed (n=126 in high acetylcholine group; n=126 in low acetylcholine group). P-value calculated by the log-rank test [file 12890_2024_2856_MOESM3_ESM.docx]

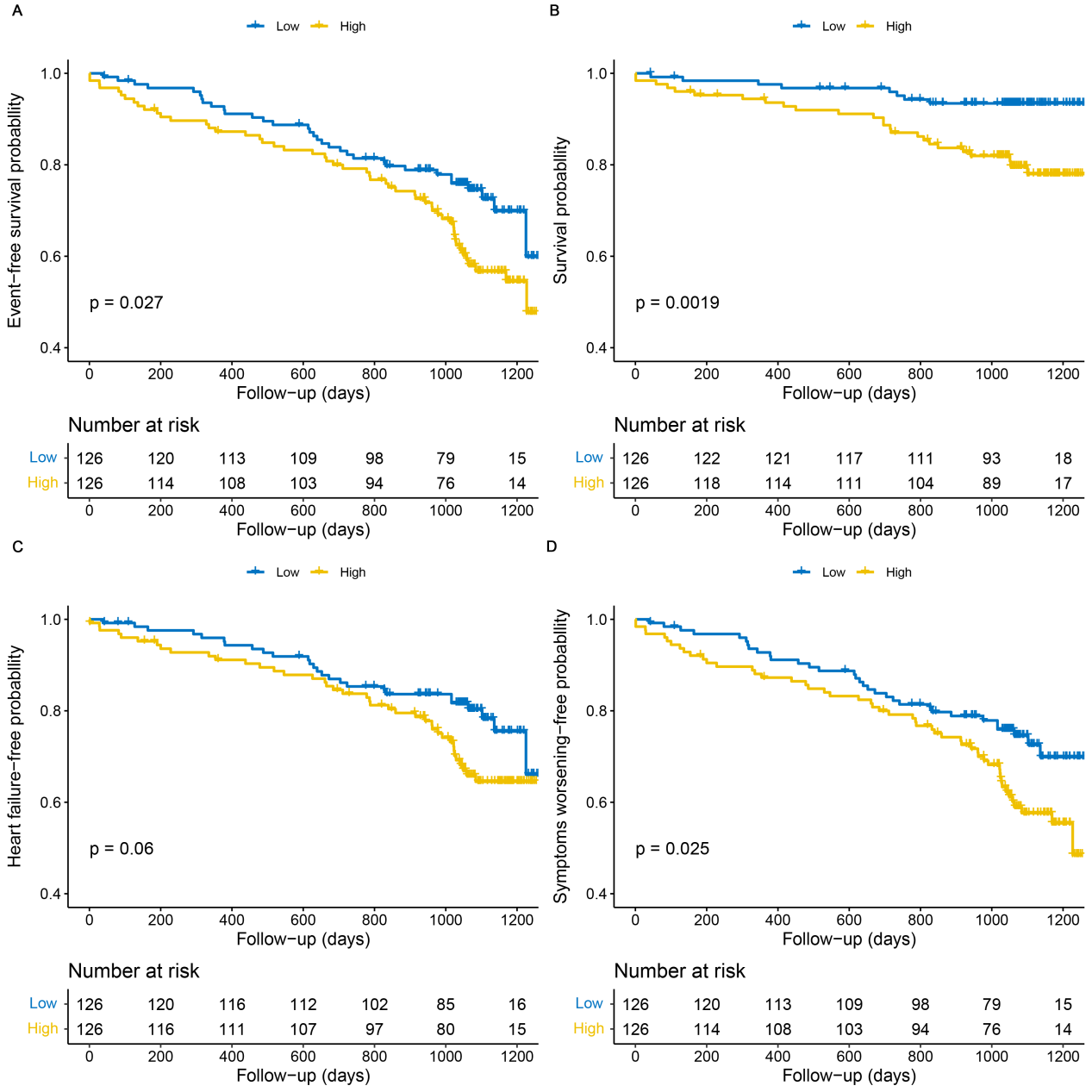


**Figure S1. Kaplan-Meier analysis for the incidence of composite outcome events(A), death (B), heart failure (C), and symptoms worsening (D) in patients with high and low acetylcholine levels after propensity score matching.** Two hundred and fifty two patients with PH were analyzed (n=126 in high acetylcholine group; n=126 in low acetylcholine group). ***P***-value calculated by the log-rank test.
